# Mobilization of Hematopoietic Stem and Progenitor Cells during Dengue Virus Infection

**DOI:** 10.3390/ijms232214330

**Published:** 2022-11-18

**Authors:** Irwin Puc, Tzu-Chuan Ho, Yu-Wen Chien, Sia-Seng Tan, Yu-Cin Fong, Yi-Ju Chen, Sheng-Hsuan Wang, Yun-Hsuan Li, Chun-Hong Chen, Po-Lin Chen, Guey-Chuen Perng, Jih-Jin Tsai

**Affiliations:** 1 Institute of Basic Medical Sciences, College of Medicine, National Cheng Kung University, Tainan 701401, Taiwan; 2 Department of Public Health, College of Medicine, National Cheng Kung University, Tainan 701401, Taiwan; 3 Department of Occupational and Environmental Medicine, National Cheng Kung University Hospital, College of Medicine, National Cheng Kung University, Tainan 701401, Taiwan; 4 Department of Microbiology and Immunology, College of Medicine, National Cheng Kung University, Tainan 701401, Taiwan; 5 National Institute of Infectious Diseases and Vaccinology, National Health Research Institutes, Zhunan 350401, Taiwan; 6 National Mosquito-Borne Diseases Control Research Center, National Health Research Institutes, Zhunan 350401, Taiwan; 7 Department of Internal Medicine, College of Medicine, National Cheng Kung University Hospital, Tainan 701401, Taiwan; 8 Tropical Medicine Center, Kaohsiung Medical University Hospital, Kaohsiung 807378, Taiwan; 9 Division of Infectious Diseases, Department of Internal Medicine, Kaohsiung Medical University Hospital, Kaohsiung 807378, Taiwan; 10 School of Medicine, College of Medicine, Kaohsiung Medical University, Kaohsiung 807378, Taiwan

**Keywords:** dengue virus, dengue, DENV infection, hematopoietic stem and progenitor cells (HSPCs), HSPCs mobilization

## Abstract

Hematopoietic stem and progenitor cells (HSPCs) mobilization is the movement of HSPCs from the bone marrow to the peripheral blood or tissue induced by stress. HSPC mobilization is a well-known response to protect the host during infection through urgent differentiation of HSPCs to immune cells. Dengue virus (DENV) infection is known to cause stress in infected humans and the mobilizing capacity of HSPCs during DENV infection in affected patients has not been fully investigated. Here, we investigated whether DENV infection can induce HSPC mobilization and if the mobilized HSPCs are permissive to DENV infection. White blood cells (WBCs) were collected from dengue patients (DENV+) and healthy donors and analyzed by flow cytometry and plaque assay. Elevated HSPCs levels were found in the WBCs of the DENV+ group when compared to the healthy group. Mobilization of HSPCs and homing markers (skin and gut) expression decreased as the patients proceeded from dengue without symptoms (DWoWS) to severe dengue (SD). Mobilizing HSPCs were not only permissive to DENV infection, but infectious DENV could be recovered after coculture. Our results highlight the need for further investigation into HSPC mobilization or alterations of hematopoiesis during viral infections such as DENV in order to develop appropriate countermeasures.

## 1. Introduction

Dengue virus (DENV) is a single positive-stranded RNA virus belonging to the *Flaviverridae* family. Its RNA transcripts can translate into three structural proteins (C, prM, and E) that compose the virion and seven nonstructural proteins (NS1, 2A/B, 3, 4A/B, 5) that are involved in viral replication [1]. DENV infection causes dengue; it is one of the most important mosquito-borne viral diseases in the world. Epidemiological studies show that the incidence of DENV infection has escalated 30-fold over the past 50 years [2], indicating that more and more people are at risk of DENV infection globally. Although the infection is asymptomatic, some patients exhibit symptoms such as fever, skin rash, diarrhea, mild bleeding, and hematological abnormalities. A few patients develop a more severe form of the disease which involves hemorrhagic fever, dengue shock syndrome, and sometimes even death [3]. 

Hematopoietic stem and progenitor cells (HSPCs) are responsible for the continuous renewal and regeneration of damaged tissues in order to maintain central hematopoiesis. During an infection, the host induces the activation and proliferation of HSPCs, which is referred to as emergency myelopoiesis [4]. HSPCs can sense the inflammatory signals and accumulate in inflamed peripheral tissue for pathogen elimination through the replenishment of tissue innate immune cells [5,6,7]. DENV infection can cause inflammation and tissue damage in dengue patients; therefore, it is essential for the host to establish an appropriate inflammatory response to clear the pathogen in order to reduce the risk of developing the disease. Several innate, inflammatory pathways and cytokines are activated at the early stages after DENV recognition to ensure resistance to the infection [3,8].

Recent studies have shown that stress-induced conditions, such as inflammation, infection, and injury can facilitate hematopoietic stem and progenitor cell (HSPC) mobilization [7,9,10,11,12]. HSPC mobilization is the movement of HSPCs from the bone marrow to the peripheral blood or tissue induced by stress. Cytokines and chemokines such as CSF3, CSF2, FLT3LG, ICAM1, CD40L, IL-33, CXCL8, CCL3, CXCL2, and CXCL12 have been observed to enhance HSPCs mobilization in humans and animal models [12,13,14,15]. Moreover, significant expression of several cytokines and chemokines, such as CSF3, CSF2, CXCL8, CXCL3, and CXCL2 have been reported in the sera of dengue patients [16,17,18]. Although DENV infection is known to cause stress in infected humans, the mobilizing capacity and infectivity of HSPCs during DENV infection in affected patients remain ill-defined [19]. Although few groups have reported that HSPCs can be infected by dengue, these studies consist mainly of experiments involving in vitro DENV infection of bone marrow (BM) or human umbilical cord blood (HUCB) specimens from normal donors [20,21]. Therefore, in this study we aimed to investigate whether DENV infection can induce HSPC mobilization. We explore the infectivity of HSPCs to DENV and the tissue homing ability of HSPCs in dengue patients.

## 2. Results

### 2.1. Mobilization of HSPCs in Dengue-Infected Patients

**Figure 1:**
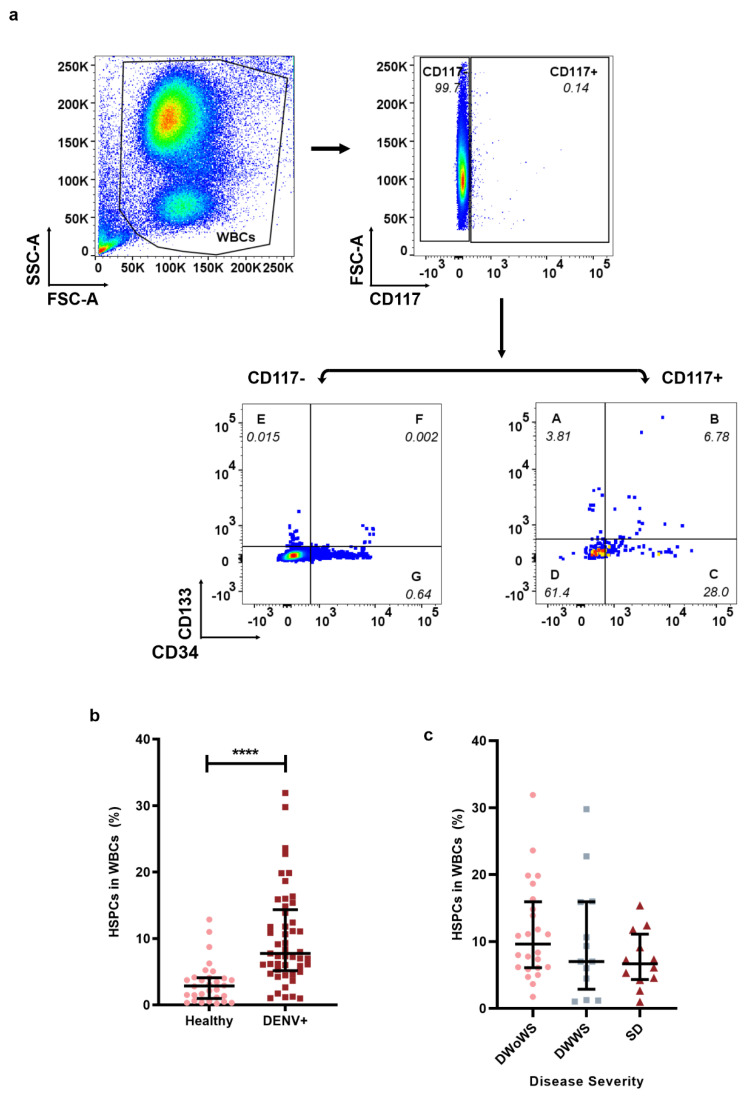
HSPCs mobilization during DENV infection. Flow cytometry was used to analyze the percentage of HSPCs in WBCs of dengue patients (DENV+, n = 49, ■) and the healthy group (Healthy, n = 30, ●). (**a**) Gating strategy for HSPCs in WBCs. (**b**) Percentage of HSPCs in WBCs of DENV+ and Healthy group. (**c**) Percentage of HSPCs in WBCs of DENV disease severity; dengue without warning sign symptoms (DWoWS), dengue with warning signs (DWWS), and severe dengue (SD). Data represented as median (IQR). *p* < 0.0001 ****.

Because DENV infection is hypothesized to induce the mobilization of HSPCs, we first explored the percentage change of circulating HSPCs in WBCs of the dengue patients’ group (DENV+) and healthy control group by flow cytometry. The gating strategy used for the HSPCs in WBCs was described in Figure 1a. Analysis of the percentage of HSPCs in WBCs was significantly higher in the DENV+ group when compared to the healthy control (7.748 (5.161–14.35) vs. 2.865 (0.9893–4.109); *p* = 0.0001) (Figure 1b). Next, we explored the mobilization percentage of HSPCs in dengue patients at the different severity of the disease. Interestingly, the mobilization of HSPCs exhibited a decreasing pattern along with the disease severity. However, there was no statistically significant difference between DWoWS, DWWS, and the SD group (9.610 (6.080–15.94) vs. 7.005 (2.861–15.93) vs. 6.660 (4.235–11.10); *p* = 0.5743), respectively (Figure 1c).

### 2.2. Homing Markers Expression in HSPCs during Dengue Infection

**Figure 2:**
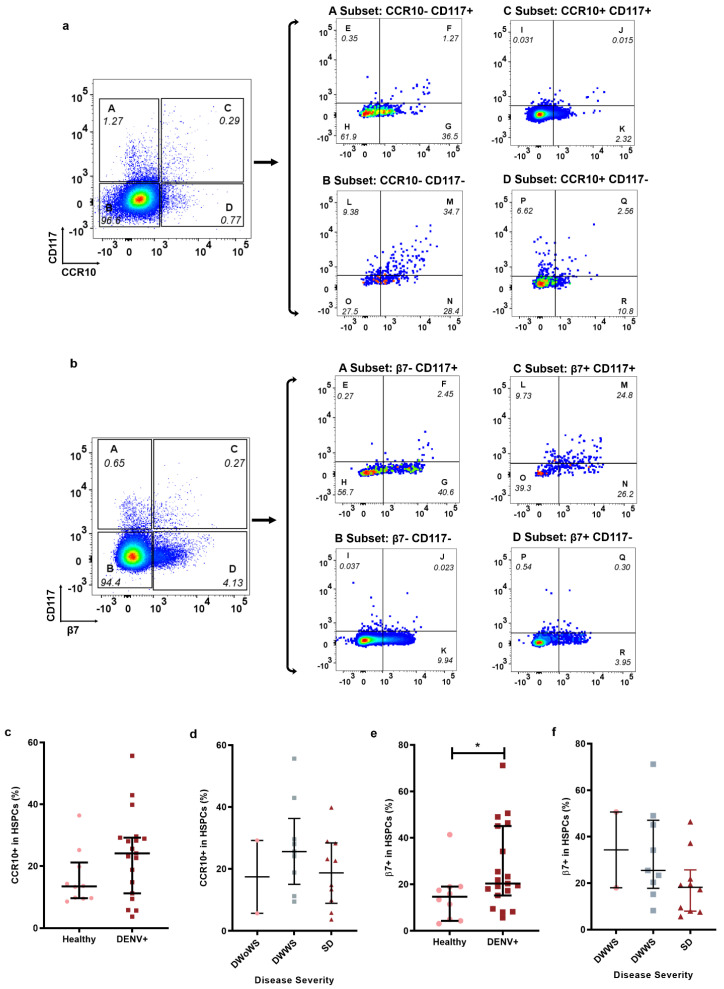
Percentage of skin- or gut-homing HSPCs in dengue patients. The percentage of skin-homing HSPCs and gut-homing HSPCs was analyzed in WBCs from healthy individuals (Healthy, n = 10, ●) and dengue patients (DENV+, n = 19, ■) using flow cytometry. (**a**) Gating strategy for skin-homing HSPCs. (**c**,**d**) The percentage of skin-homing HSPCs (CCR10+ in HSPCs). (**b**) Gating strategy for gut-homing HSPCs. (**e**,**f**) The percentage of gut-homing HSPCs (β7+ in HSPCs). c (IQR). *p* < 0.05 *.

In order to explore the homing capacity of HSPCs to the skin or gut in the peripheral blood of dengue patients, antibodies against CCR10 and β7 integrins were used to detect the expression of skin- or gut-homing molecular markers on HPSCs, because these homing makers have been well documented and widely used in previous studies [22,23,24,25]. The gating strategy for skin-homing HSPCs (CCR10+HSPCs) was described in Figure 2a. Although the percentage of HSPCs in peripheral blood of dengue patients (DENV+ group) expressing CCR10 showed a visible increase pattern compared to the healthy group, there was no significant difference between the healthy and DENV+ group (13.44 (9.624–21.18) vs. 24.12 (11.21–29.22); *p* = 0.05), probably due to the small sample size (Figure 2c). It was also noted that HSPCs expressing the homing marker CCR10 in peripheral blood of DENV patients fluctuated along with the progression of the disease severity (Figure 2d).

The gut-homing marker, β7 expression in HSPCs in peripheral blood of participants (β7+HSPCs), was gated as described in Figure 2b. The percentage of β7+ in HSPCs of peripheral blood cells was significantly higher in the DENV+ group when compared to the healthy control (20.31 (15.18–45.09) vs. (14.63 (4.709–18.93); *p* = 0.0274) (Figure 2e). Interestingly, it was also noted that HSPCs expressing the homing marker β7 in peripheral blood decreased along with the progression of the disease severity (Figure 2f).

### 2.3. HSPCs in Dengue Patients Were Permissive for DENV Infection

**Figure 3:**
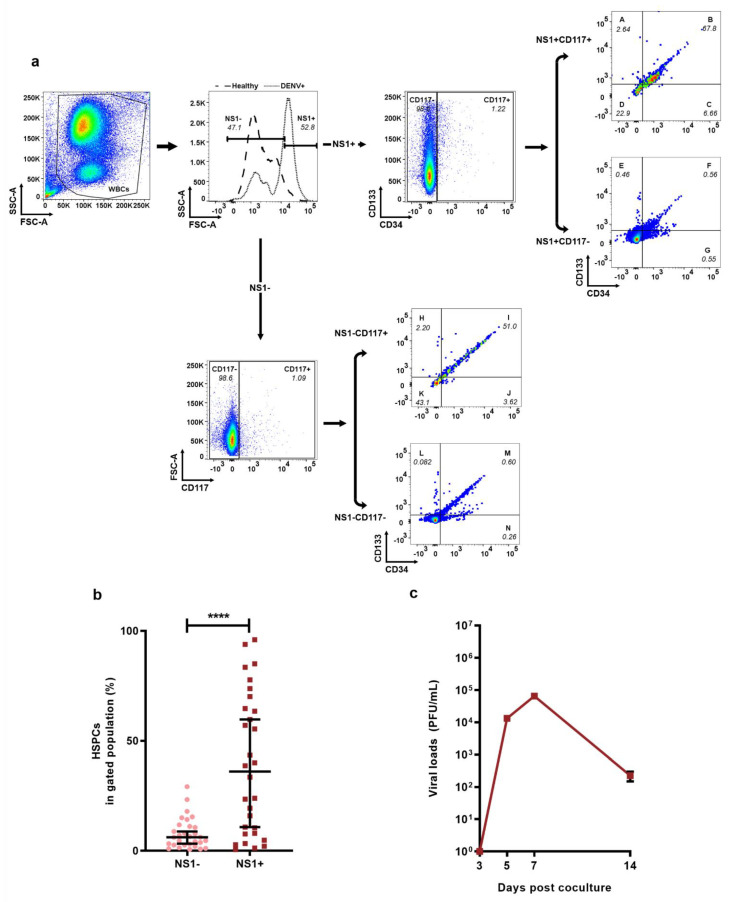
HSPCs were permissive to DENV infection. (**a**) Gating strategy of DENV-infected and -uninfected HSPCs. (**b**) Percentage of DENV-infected HSPCs (NS1+, n = 30, ■) and DENV-uninfected HSPCs (NS1−, n = 30, ●). (**c**) The kinetic titer of DENV recovered from HSPCs after coculture with Vero cells (n = 3). Data represented as median (IQR). *p* < 0.0001 ****.

To further evaluate whether the HSPCs population could be infected by DENV in vivo, we utilized flow cytometry to analyze the percentage of HSPCs in DENV-infected WBCs and DENV-uninfected WBCs. The gating strategy used was described in Figure 3a. Infected cells were detected by intracellular labeling of a monoclonal antibody directed to DENV NS1; previous studies have also used NS1 to confirm dengue virus infection in blood cells [26,27]. In summary, we first explored and overlaid the NS1 profile of both the DENV+ group and healthy group in a histogram plot to define the NS1+/− subset in WBCs of the DENV+ group, the HSPCs population was gated on the NS1+ and NS1− subsets. The detailed gating strategy was described in the Materials and Methods section. Analysis revealed that the percentage of HSPCs was significantly higher in NS1+ WBCs group when compared to the NS1− WBCs group (36.11 (7.885–66.00) vs. (6.162 (2.629–11.27); *p* = 0.0001) (Figure 3b).

To investigate whether infectious DENV can be recovered from HSPCs, HSPCs were sorted out from the peripheral blood cells of a dengue patient and cocultured with Vero cells. The kinetic curve was plotted in Figure 3c. The results demonstrated that a low titer (50 PFU/mL) of DENV could be detected on day 3 post-coculture, followed by a sudden rise in DENV titer on days 5 and 7 and maintained a steady decrease up to day 14.

## 3. Discussion

The ability of the bone marrow (BM) to produce an estimated half a trillion new cells per day is seen as a highly dynamic process, and it is due to the proliferation and differentiation of HSPCs residing in BM [28,29]. Under normal conditions these HSPCs remain in specialized niches within the bone marrow but mobilize during an infection, injury, or stress-induced condition. HSPCs are responsible for the continuous renewal and regeneration of damaged tissues in order to maintain central hematopoiesis, and, more importantly, to produce almost all mature blood and immune cells [4,5,9,30]. Although the mobilization of HSPCs in bacterial infections or systemic inflammation has been reported, HSPCs mobilization or alterations of hematopoiesis to viral infections such as DENV have received far less attention [31]. In fact, to the best of our knowledge this is the first time the mobilization of HSPCs in DENV infected patients has been studied. 

In this study, we compared specimens collected from DENV−2-infected patients (DENV+) and healthy donors, with the aim of investigating whether DENV infection can induce HSPC mobilization. We explore the infectivity of HSPCs to DENV and the tissue homing ability of HSPCs in dengue patients. The analyzed data acquired from the flow cytometry demonstrated that HSPCs mobilization was induced upon DENV infection (Figure 1b), which is consistent with previous studies in other stress-induced conditions and infections, such as *Escherichia coli* strain K12, *Listonella anguillarum*, and septic shock [7,9,32]. Interestingly, we observed that the mobilization of HSPCs during DENV infection decreased as the disease proceeded to a more severe form (Figure 1c). Over the years, galectins have been associated with dengue infection; they are a family of lectins that are released under stress conditions such as infection [33]. Although they are very simple molecules, they have been observed to perform a wide variety of functions such as cell proliferation, cell adhesion, cytokine/chemokine production, signal transduction, and mobilization [33,34]. Moreover, recent reports have detected high levels of galectin-9 in DENV-infected patients which not only correlated with high DENV titer, but also with the disease severity [35]. Evidence has also demonstrated that HSPCs have reduced hematopoietic output in septic shock patients and other viral infections [31,32]. Whether the decreased mobilization of HSPCs is a cause or consequence of the disease severity or small molecules like galectin remains ill-defined because HSPC mobilization in DENV has not been explored. BM suppression is one of the hallmarks of severe DENV infection in patients affecting all cell populations in the bone marrow including HSPCs, and direct viral targeting of the BM has been speculated. Clinically, it has been observed that BM suppression occurs two to three days before thrombocytopenia and leukopenia (reduction of white blood cells and platelets, respectively) [36]. The critical period for dengue in progression to severe conditions falls between four and six days after the onset of the illness. Therefore, we speculated that because HSPCs mobilization has been seen as part of the process of our innate immunity, it might occur in the early and critical period and possibly vary in hosts with different immunity. However, the role of HSPCs mobilization when the patient approaches recovery is unclear and cannot be inferred from our study. Even though this has been a common phenomenon observed clinically as the patients progress from nonsevere to severe DENV infection, the exact mechanism remains unclear. Emerging evidence has demonstrated that an aberrant immune response in the initial stages of infection leads to a more severe form of the disease and may be lethal for the host. Previous studies investigating BM suppression in infected humanized mice provided evidence that DENV reduces megakaryocytes (Mks) and their progenitor cells [37]. Given the fact that Mks are derivatives of HSPCs and responsible for the formation of thrombocytes and platelets, it is possible that during DENV infection, the decreased mobilization of HSPCs, as observed in this study, may impact hematopoietic cells that potentially have an effect on the disease severity.

Cytokines and chemokines such as CSF 3, CSF2, FLT3LG, ICAM1, CD40L, IL-33, CXCL8, CCL3, CXCL2, and CXCL12 have been observed to enhance HSPCs mobilization and accumulate in inflamed peripheral tissue for pathogen elimination through the replenishment of tissue innate immune cells [4,12,13,14,15,38]. We previously provided evidence that cytokine dysfunction reflected by cytokine storm contributes to the disease severity. We demonstrated that cytokines such as ICAM1, CD40L, IL-4, and IL-33 decreased in the early illness phase of the disease and remained low as the illness progressed [39]. Therefore, it can be speculated that the reason why we observed a decrease in the mobilization of HSPCs is that the patients proceeded from dengue without warning sign symptoms (DWoWS) to the more severe form of the disease, severe dengue (SD) (Figure 1c). 

The homing of HSPCs plays an important role in the maintenance of central hematopoiesis, which is the recruitment and homing of stem and progenitor cells to damaged tissue or site of infection. Our results demonstrated that there was no significant difference in the percentage of HSPCs expressing skin-homing marker CCR10 in healthy patients and DENV-infected patients (Figure 2c,d). However, there was a significant difference in the percentage of HSPCs expressing gut-homing marker β7 between healthy patients and DENV-infected patients. Moreover, there was a decrease in the expression levels as the disease progressed to a more severe form (Figure 2e,f). However, we did not investigate whether DENV infection would cause dysfunction in the differentiation of these HSPCs to immune cells or other cell populations, or whether it would affect the homing capacity. Very few studies have reported the dysfunction in the homing capacity and differentiation of HSPCs. For example, direct infection of HSPCs derived from HUCB with DENV can hinder the differentiation of HSPCs to platelet progenitor cells. Likewise, the number of myeloid cells is reduced in HBM infected with DENV [20,21,40]. Other viral and bacterial infections can affect the differentiation of HSPCs, which impairs the ability of HSPCs mobilization. The colony-forming unit assay for CD34+ HSPCs isolated from HBM and peripheral blood have shown that the differentiation of megakaryocytes is largely inhibited in patients with human immunodeficiency virus [41], which has been reasoned due to the generation of neutrophils instead [42]. However, defective production of common myeloid progenitors and granulocyte-monocyte progenitors is also found in the lethal sepsis animal model [43]. Taken together, these findings suggest that much more work is needed to completely understand the role of BM suppression in the pathogenesis of dengue. 

Traditionally, DENV is transmitted from person to person through the bite of an infected female mosquito. However, unconventional transmissions like blood transfusion, solid organ transplantation, and hematopoietic stem cell transplantation have been reported in recent years [44,45,46]. Recent evidence confirms that DENV can be transmitted through HSPCs transplantation and that HSPCs from BM or HUCB are infectable by DENV [21,40]. These reports point out that DENV could be disseminated through HSPC transplantation, triggering concern about the safety in the practice of HSPC transplantation in dengue-prone regions [47,48]. However, these reports are mainly ex vivo investigations with HSPCs from BM or HUCB being infected with DENV; whether infectious DENV could be recovered from sorted HSPCs in peripheral blood of dengue patients remains to be verified. Our data showed that not only HSPCs were infectable by DENV, but more importantly infectious DENV could be recovered from sorted HSPCs from peripheral blood of dengue patients after coculturing with Vero cells (Figure 3b,c). Our data showed that the infectious DENV could be recovered from sorted HSPCs from peripheral blood of dengue patients (Figure 3c), which is consistent with a previous report on the permissiveness of HSPCs to DENV in adult BM [20]. This result highlights the need for DENV screening of hematopoietic stem cell donors in tropical and subtropical countries where dengue is endemic.

There are several limitations to our study. Our study involves a low number of volunteers in the healthy control group and DENV+ group. We were unable to identify the previous infection history of the patients to see whether this was a secondary infection. All patients were adults and infected with DENV serotype 2, restricting our ability to compare differences with another serotype. Hence, our results may only be applicable to adults affected with DENV serotype 2 and may differ in pediatric patients or individuals infected with other serotypes. However, DENV serotype 2 is considered the most severe. Various studies have reported that DENV serotype 2 exhibits a higher proportion of severe cases and is more readily transmitted than the other three serotypes in various communities [49,50]. Therefore, future investigations with different serotypes, demographic characteristics, comorbidities, immunological status, and disease severity could contribute to elucidating the role of HSPC mobilization and influence in the progression of severe dengue.

## 4. Materials and Methods

### 4.1. Ethical Statement and Study Cohort

In this study, a total of 79 patients were enrolled to observe and analyze their blood to try and determine whether DENV infection could induce HSPCs mobilization. Samples with written informed consent were obtained from dengue patients (DENV+) as they were admitted to National Cheng Kung University Hospital (NCKUH) and Kaohsiung Medical University Hospital (KMUH) during the 2015 Taiwan dengue outbreak. To maintain the confidentiality of samples, clinical data were recorded as donor numbers in the report. Patients that were laboratory-confirmed positive for DENV by real-time PCR (polymerase chain reaction) were classified as dengue patients (DENV+) group, which consisted of 49 patients. All DENV+ patients were DENV serotype 2 (DENV−2). The healthy control group consisted of 30 patients. Patients were further classified according to the 2009 WHO dengue classification guidelines into dengue without warning sign symptoms (DWoWS) which consisted of 24 patients; dengue with warning signs which consisted of 13 (DWWS) and severe dengue (SD) which consisted of 12 patients. Data characteristics of the patient age, donor number, serological tests, and disease severity classification in this study were provided in Supplementary Table S1. These samples were collected within seven days during the acute stage. This study was approved by the Institutional Review Boards of National Cheng Kung University Hospital (IRB #B-ER-104-178) and by Kaohsiung Medical University Hospital (KMUHIRB-960195).

### 4.2. White Blood Cells Preparation

Fresh blood was drawn from the patients and transferred into 50-mL tubes. The blood was then centrifuged at 1000× *g* for 8 min at 4 °C. After centrifugation, the top plasma layer was carefully collected and stored at −80 °C for rapid immunochromatographic tests. After the removal of the blood plasma, 10 mL of red blood cells (RBCs) lysis buffer (#158904, Qiagen, Valencia, CA, USA) was added into the centrifuge tube and mixed for 10 min to remove the RBCs. The mixture was centrifuged at 300× *g* for 8 min at 4 °C and the supernatant was discarded. This process was repeated twice to ensure the complete removal of the RBCs. After centrifugation, the white blood cell (WBC) pellet was collected.

### 4.3. Fluorochrome Conjugated DENV NS1 Antibody

Following the manufacturer’s guideline for Alexa FluorTM 647 Microscale Protein Labeling kit (ThermoFisher Scientific Inc., Waltham, MA, USA), 60 µg of DENV NS1 antibody (clone GIE9, CTK Biotech, Poway, CA, USA) was mixed with 1 µL of fluorescent dye, Alexa 647, in sodium bicarbonate solution (pH~8.3) at room temperature (RT) for 15 min, avoiding light. The final volume of the mixture equaled 50 µL. The reaction mixture was added to the resin bed constructed with resin gel and centrifuged at 16,000× *g* for 15 s to separate the labeled NS1 antibody from the unreacted dye. Alexa 647 conjugated NS1 antibodies were stored at 4 °C. Intracellular DENV binding ability of the NS1 conjugated antibody was confirmed in DENV-infected Meg-01 cells (Supplementary Figure S1).

### 4.4. Flow Cytometric Analysis

In order to investigate the kinetics of HSPC mobilization and circulation during dengue virus infection, we used different combinations of antibodies specific for hematopoietic stem cell markers. Because the CD133+ or /and CD117+ or /and CD34+ population phenotype has been extensively used to identify HSCs in numerous studies, we decided to include those phenotypes in our panel. A total of 1 × 10^6^ WBCs from healthy and DENV+ groups were stained with fluorochrome-labeled antibodies. For HSPCs staining, anti-CD133-PE (clone 293C, Miltenyi Biotec, San Jose, CA, USA), CD117-PerCP-Cy5.5 (clone YB5.B8, BD Biosciences, Franklin Lakes, NJ, USA), or APC-Cy7 (clone 104D2, BioLegend, San Diego, CA, USA), and CD34-PE-Cy7 (clone 581, BD Biosciences, Franklin Lakes, NJ, USA) were used to stain the cells at 4 °C for 1 h. The stained cells were washed by centrifuging with 3 mL of surface staining buffer (1% BSA and 0.1% sodium azide in PBS) at 300 g for 8 min. For detecting DENV in HSPCs, stained cells were then permeabilized with 100 µL of FOXP3 Fix/Perm Buffer (#421403, BioLegend, San Diego, CA, USA) at RT for 20 min. Permeabilized WBCs were stained with 0.2 µL of anti-DENV NS1 Alexa 647 antibody in 100 µL of intracellular staining buffer (1% BSA, 0.1% sodium azide, and 0.1% saponin in PBS) for 1 h at 4°C. For the homing marker staining in HSPCs, WBCs were stained with anti-CCR10-BB515 antibody (clone 1B5, BD Biosciences, Franklin Lakes, NJ, USA), integrin β7-APC antibody (clone FIB504, BD Biosciences, Franklin Lakes, NJ, USA), and antibodies against HSPCs as above described for 1 h at 4 °C. After staining, 3 mL of surface staining buffer was used to wash the stained cells by centrifuging at 300× *g* for 8 min. The cell pellet was then resuspended with 300 µL of surface staining buffer. Data acquisition was performed on a Flow Cytometry Fortessa X20 Cytometer (BD LSRFortessaTM, BD Biosciences, Franklin Lakes, NJ, USA). At least 100,000 events were recorded in the mononuclear cell gate set on the FSC/SSC morphological plot. The acquired data were analyzed and visualized by using FlowJo v10 software (BD Biosciences, Franklin Lakes, NJ, USA).

### 4.5. Gating Strategy for HSPCs, Homing HSPCs, and DENV Infected or Uninfected HSPCs

To easily determine the percentage of the different populations by using dot blot analysis in FlowJo v10, the WBC population was separated into seven subsets since the population cannot be directly gated together in one dot blot. The seven different subsets were, CD133+CD117+CD34−, CD133+CD117+CD34+, CD133−CD117+CD34+, CD133−CD117+CD34−, CD133+CD117−CD34−, CD133+CD117−CD34+, and CD133−CD117−CD34+. Each subset was gated on WBCs by analysis of dot blot. The gating strategy of HSPCs was described in Figure 1a. The WBCs were gated on forward scatter/side scatter (FSC/SSC) dot blot. CD117+WBCs and CD117−WBCs were gated on WBCs. The subsets of CD133+CD117+CD34−, CD133+CD117+CD34+, CD133−CD117+CD34+, CD133−CD117+CD34− were gated on CD117+ WBCs and successively labeled as A, B, C, and D on CD133/CD34 dot blot in Figure 1a. The subsets of CD133+CD117−CD34−, CD133+CD117−CD34+, and CD133−CD117−CD34+ were gated on CD117−WBCs and labeled as E, F, and G in Figure 1a. The percentage of HSPCs was the sum of these seven subsets in WBCs.

The gating strategy of skin-homing HSPCs (CCR10+ in HSPCs) was described in Figure 2a. The four subsets consisted of CD117+/− cells and CCR10+/− cells that were gated on WBCs, and then seven subsets of HSPCs were gated on four populations. The percentage of HSPCs was the total sum of expression in the different subsets denoted as E, F, G, H, I, J, K, L, M, N, O, P, Q, and R. The percentage of CCR10+HSPCs in the WBCs was the total sum of subsets denoted as L, M, N, O, P, Q, and R in Figure 2a. The percentage of skin-homing HSPCs (CCR10+ in HSPCs) was equal to the percentage of CCR10+HSPCs in the WBCs derived by that of HSPCs. The gating strategy of gut-homing HSPCs (β7+ in HSPCs) followed the same gating strategy used in skin-homing HSPC gating. The percentage of gut-homing HSPCs (β7+ in HSPCs) was also equal to the percent of β7+HSPCs in the WBCs derived from that of HSPCs, the gating strategy of HSPCs was described in Figure 2b.

The gating strategy of DENV in HSPCs was shown in Figure 3a. Compared with NS1 profiling of healthy donors (dashed line), the dengue NS1 profiling (solid line) was gated on NS1+ and NS1− respectively, then gated out the seven subsets of HSPCs. The seven subsets of HSPCs in the NS1+ population were separately labeled as subsets of A, B, C, D, E, F, and G. In the NS1− population, subsets H, I, J, K, L, M, and N were separately labeled. The percentage of DENV-infected HPSCs was the sum of expression in the subsets of A, B, C, D, E, F, and G. The percentage of DENV-uninfected HSPCs was the sum of expression in the subsets of H, I, J, K, L, M, and N.

### 4.6. HSPCs Isolation

Microbead and isolation kits by MACS from Miltenyi Biotec were used to isolate the HSPCs from the blood cells of dengue patients. Following the manufacturer’s guideline, a total of 7 × 10^6^ blood cells were mixed and incubated with each microbead-conjugated antibodies against human CD133 (# 130-097-049), CD117 (#130-091-332), and CD34 (#130-097-047) for 40 min at 4 °C and inverted gently every 10 min during the incubation period. After incubation, it was washed with 1 mL of MACS buffer (#130-091-221) by centrifugation at 300× *g* for 10 min. After 10 min, the cells were resuspended in 500 µL of MACS buffer and proceeded with the isolation of HSPCs using the MS column (# 130-042-201) in the magnetic field of the MACS separator (#130-042-102).

### 4.7. Virus Titration

Viral loads of DENV in cocultured supernatants were titrated by the standard viral plaque assay as previously described [51]. In brief, BHK21 C-13 cells (ATCC, Manassas, VA, USA) were seeded in six-well plates, containing 8 × 105 cells per well with 3 mL of 5% DMEM for 16 to 20 h. A total of 100 µL of cocultured supernatants were 10-fold sequentially diluted up to 1000-fold with 2% DMEM. The old medium in the culture plate was removed, and then 400 µL of the diluted solution was added to each well. The diluted samples were incubated with BHK21 C-13 cells for 2 h and shaken every 15 min during the incubation period. Then diluents were aspirated, and 3 mL of overlay medium (1% methylcellulose in 2% DMEM at pH 7.4–7.6) was laid over the wells. Plaques were visualized by staining with 1% crystal violet and counted after 6 to 7 days.

### 4.8. Statistical Analysis

All raw data were stored in a computerized database (MS Excel 2016, Microsoft, Redmond, WA, USA). Statistical analysis and data visualization were performed by using R-Studio v1.2.5042 (RStudio: Integrated Development for R. RStudio, Boston, MA, USA) and GraphPad Prim v7 (GraphPad Software, San Diego, CA, USA). Data are presented as n (%) or median (IQR). Comparisons between the healthy control and dengue group were performed by Mann–Whitney U test. Scatter plots were used to visualize the distribution of the data within the two groups. A *p*-value of < 0.05 was considered significantly different by statistical analysis. General statistics in this study were provided in Supplementary Table S2.

## 5. Conclusions

In conclusion, these observations from previous research and our work further provide new insight and evidence that DENV infection can induce HSPC mobilization. The mobilized HSPCs were not only permissive to DENV infection, but infectious DENV could be recovered after coculture. Further investigation into HSPC mobilization or alterations of hematopoiesis during viral infections, such as DENV infection, is needed to better understand the invasion, transmission, and pathogenesis of DENV and to develop appropriate countermeasures.

## Data Availability

All data generated during this study are included in this published article and are available from the corresponding author upon reasonable request.

## Supplementary Materials

The following supporting information can be downloaded at: https://www.mdpi.com/article/10.3390/ijms232214330/s1.
