# Supplementary material for: Mobilization of Hematopoietic Stem and Progenitor Cells during Dengue Virus Infection
Source: Int J Mol Sci. 2022 Nov 18;23(22):14330. doi: 10.3390/ijms232214330 (PMC9699116; doi:10.3390/ijms232214330)

International Journal of *Molecular Sciences*

MDPI

# *Supplementary Material*

**Supplementary Table**

**Supplementary Table S1. Study Cohort Summary and laboratory parameters.** DENV patients were divided into three groups according to the day of illness and clinical symptoms following the 2009 WHO dengue classification scheme: dengue without warning sign symptoms (DWoWS, n=24), dengue with warning signs (DWWS, n=13) and severe dengue (SD, n=12). Healthy (Healthy, n= 30).

| Dengue Group | | | | | | | |
|---|---|---|---|---|---|---|---|
| Donor No. (n=49) | Viral load (from viral culture) | WHO Classification | Gender | Age | Duo Dengue Ag-IgG/IgM Rapid Test NS1 | IgM | IgG |
| Donor 1 | - | DWoWS | M | 27 | + | - | - |
| Donor 2 | + | DWWS | F | 72 | + | - | - |
| Donor 3 | + | DWWS | M | 75 | + | - | - |
| Donor 4 | + | DWoWS | M | 39 | - | - | - |
| Donor 5 | + | DWoWS | M | 27 | + | - | - |
| Donor 6 | - | DWoWS | F | 23 | + | - | - |
| Donor 7 | + | DWWS | M | 69 | + | - | - |
| Donor 8 | + | SD | M | 69 | + | - | - |
| Donor 9 | + | SD | M | 80 | + | + | + |
| Donor 10 | + | DWWS | M | 75 | + | - | - |
| Donor 11 | + | DWWS | M | 80 | + | + | + |
| Donor 12 | + | DWWS | F | 72 | + | - | - |
| Donor 13 | + | DWWS | M | 75 | + | + | - |
| Donor 14 | - | SD | M | 69 | + | + | + |
| Donor 15 | - | SD | M | 69 | + | + | + |
| Donor 16 | + | DWWS | M | 75 | + | - | - |
| Donor 17 | - | SD | M | 80 | + | + | + |
| Donor 18 | - | DWWS | M | 28 | - | + | + |
| Donor 19 | - | SD | M | 80 | - | + | + |
| Donor 20 | - | SD | M | 28 | - | + | + |
| Donor 21 | + | DWWS | F | 28 | - | - | - |
| Donor 22 | + | DWWS | M | 75 | + | + | + |
| Donor 23 | - | SD | M | 69 | - | + | + |

| | | | | | | | |
|---|---|---|---|---|---|---|---|
| Donor 24 | - | DWoWS | M | 27 | + | + | + |
| Donor 25 | - | SD | M | 80 | - | + | + |
| Donor 26 | - | SD | M | 28 | - | + | + |
| Donor 27 | + | DWWS | F | 72 | - | + | - |
| Donor 28 | - | DWWS | M | 75 | - | + | + |
| Donor 29 | - | SD | M | 69 | - | + | + |
| Donor 30 | - | SD | M | 80 | - | + | + |
| Donor 31 | + | DWoWS | F | 65 | + | - | - |
| Donor 32 | + | DWWS | M | 58 | - | + | + |
| Donor 33 | + | DWoWS | M | 25 | + | - | + |
| Donor 34 | + | DWoWS | M | 65 | + | - | - |
| Donor 35 | - | DWoWS | F | 70 | + | + | + |
| Donor 36 | - | DWoWS | F | 74 | - | - | - |
| Donor 37 | + | DWoWS | M | 24 | + | + | + |
| Donor 38 | + | DWoWS | M | 43 | + | + | - |
| Donor 39 | - | DWoWS | F | 24 | + | - | + |
| Donor 40 | + | DWoWS | F | 27 | + | + | - |
| Donor 41 | - | DWWS | F | 52 | + | + | + |
| Donor 42 | - | DWoWS | M | 56 | - | + | + |
| Donor 43 | + | DWoWS | F | 72 | + | + | + |
| Donor 44 | + | DWoWS | M | 61 | + | + | - |
| Donor 45 | - | DWoWS | F | 66 | + | + | + |
| Donor 46 | + | DWWS | F | 31 | + | + | - |
| Donor 47 | - | DWoWS | M | 49 | + | + | + |
| Donor 48 | + | DWoWS | M | 24 | + | - | + |
| Donor 49 | - | DWoWS | F | 31 | - | + | + |

**Healthy Group**

| Donor No. (n=49) | Viral load (from viral culture) | WHO Classification | Gender | Age | Duo Dengue Ag-IgG/IgM Rapid Test | | |
|---|---|---|---|---|---|---|---|
| | | | | | NS1 | IgM | IgG |
| Donor 1 | - | Healthy | F | 56 | - | - | - |
| Donor 2 | - | Healthy | F | 57 | - | - | - |
| Donor 3 | - | Healthy | F | 44 | - | - | - |
| Donor 4 | - | Healthy | M | 44 | - | - | - |
| Donor 5 | - | Healthy | M | 32 | - | - | - |
| Donor 6 | - | Healthy | F | 33 | - | - | - |
| Donor 7 | - | Healthy | M | 40 | - | - | - |
| Donor 8 | - | Healthy | M | 23 | - | - | - |
| Donor 9 | - | Healthy | F | 21 | - | - | - |
| Donor 10 | - | Healthy | M | 22 | - | - | - |

| | | | | | | | |
|---|---|---|---|---|---|---|---|
| Donor 11 | - | Healthy | F | 22 | - | - | - |
| Donor 12 | - | Healthy | M | 24 | - | - | - |
| Donor 13 | - | Healthy | F | 21 | - | - | - |
| Donor 14 | - | Healthy | F | 56 | - | - | - |
| Donor 15 | - | Healthy | F | 56 | - | - | - |
| Donor 16 | - | Healthy | F | 68 | - | - | - |
| Donor 17 | - | Healthy | F | 60 | - | - | - |
| Donor 18 | - | Healthy | F | 41 | - | - | - |
| Donor 19 | - | Healthy | F | 68 | - | - | - |
| Donor 20 | - | Healthy | F | 63 | - | - | - |
| Donor 21 | - | Healthy | F | 49 | - | - | - |
| Donor 22 | - | Healthy | F | 68 | - | - | - |
| Donor 23 | - | Healthy | F | 41 | - | - | - |
| Donor 24 | - | Healthy | F | 45 | - | - | - |
| Donor 25 | - | Healthy | F | 50 | - | - | - |
| Donor 26 | - | Healthy | F | 46 | - | - | - |
| Donor 27 | - | Healthy | F | 54 | - | - | - |
| Donor 28 | - | Healthy | M | 48 | - | - | - |
| Donor 29 | - | Healthy | F | 34 | - | - | - |
| Donor 30 | - | Healthy | F | 60 | - | - | - |

**Supplementary Table S2. General Statistics.** WHO dengue 2009 classification scheme: dengue without warning sign symptoms (DWoWS, n=24), dengue with warning signs (DWWS, n=13) and severe dengue (SD, n=12). Healthy (Healthy, n= 30). Data are presented as n (%) or median (IQR). A p-value of < 0.05 was considered significantly different by statistical analysis.

| | Healthy | DENV+ | P-value |
|---|---|---|---|
| **Number** | 30 (37.9) | 49 (62.1) | |
| **Age** | 45 (33 - 56) | 66 (28 - 74) | |
| **Gender** | M (7) F (23) | M (35) F (14) | |
| **HSPCs** | | | |
| *HSPCs in WBCs* | 2.865 (0.9893 - 4.109) | 7.748 (5.161 - 14.35) | <0.0001 |
| *NS1 +/- HSPCs in WBCs* | 6.162 (2.629 - 11.27) | 36.11 (7.885 - 66.00) | <0.0001 |
| **Homing Markers** | | | |
| *CC10+ HSPCs in WBCs* | 13.44 (9.624 - 21.18) | 24.12 (11.21 - 29.22) | 0.1507 |
| *β7 + in HSPCs in WBCs* | 14.63 (4.709 - 18.93) | 20.31 (15.18 - 45.09) | 0.0274 |

| **HSPCs in Disease Severity** | Number | *Median (IQR)* | P-value |
|---|---|---|---|
| *DWoWS* | 24 (49) | 9.610 (6.080 - 15.94) | 0.3866 |
| DWWS | 13 (26.5) | 7.005 (2.861 - 15.93) | 0.5743 |
| *SD* | 12 (24.5) | 6.660 (4.235 - 11.10) | 0.084 |

**Supplementary Figures**

Supplementary Figure S1. Intracellular DENV binding ability of the NS1 conjugated antibody was confirmed in DENV−infected Meg−01 cells.

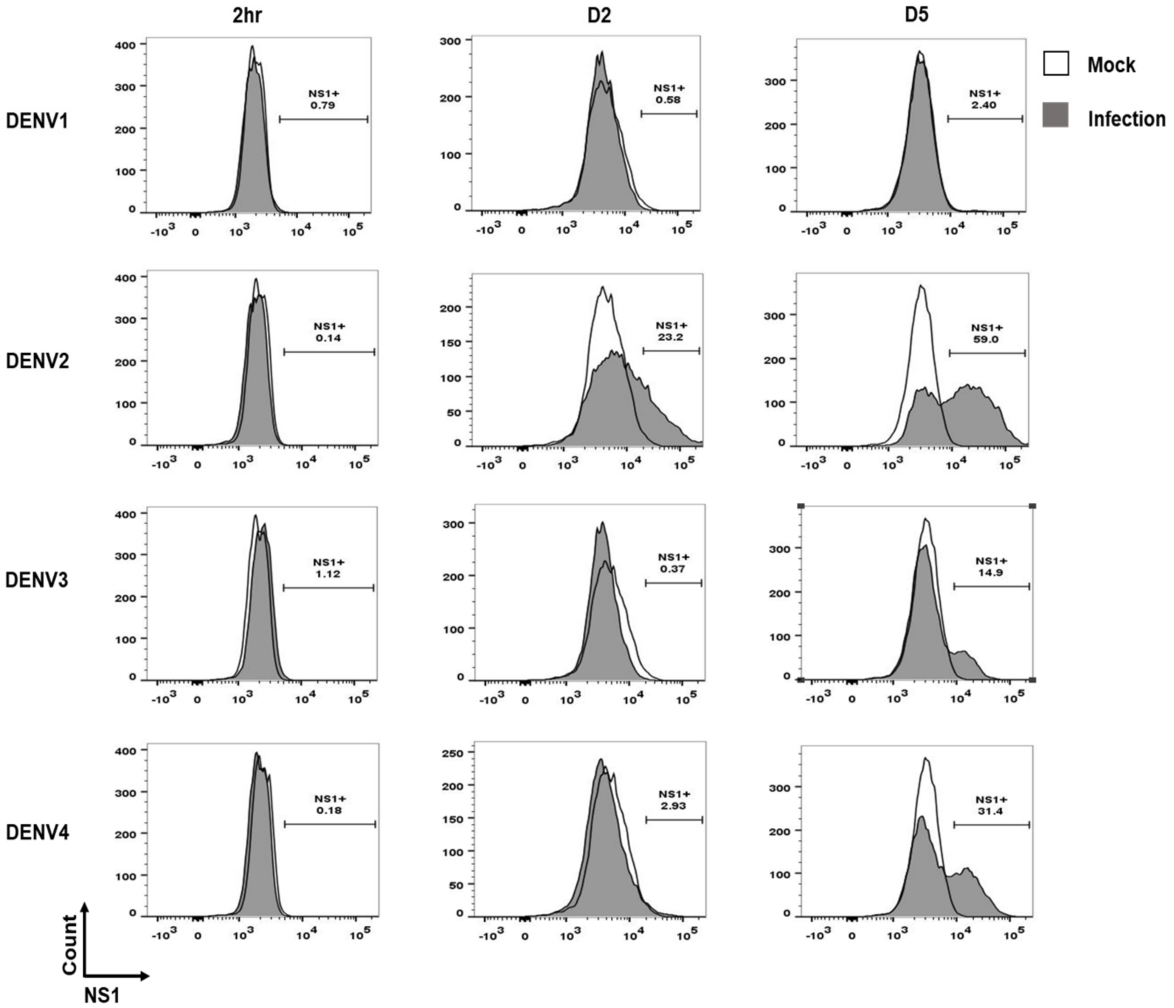

Supplement: Supplementary file 1 [file ijms-23-14330-s001.zip › ijms-1956634-supplementary.pdf]
